# Efficient piezoelectric ZnO nanogenerators based on Au-coated silica sphere array electrode

**DOI:** 10.1186/1556-276X-8-511

**Published:** 2013-12-05

**Authors:** Yeong Hwan Ko, Goli Nagaraju, Jae Su Yu

**Affiliations:** 1 Department of Electronics and Radio Engineering, Institute for Laser Engineering, Kyung Hee University, 1 Seocheon-dong, Giheung-gu, Yongin-si, Gyeonggi-do 446-701, Republic of Korea

**Keywords:** Piezoelectric nanogenerators, Zinc oxide, Silica spheres, Rough surface of top electrode

## Abstract

We reported ZnO nanorod-based piezoelectric nanogenerators (NGs) with Au-coated silica sphere array as an efficient top electrode. This electrode can readily bend the ZnO nanorods due to its enhanced surface roughness, thus resulting in more increased and regular piezoelectric charge output. Under a low external pushing force of 0.3 kgf, the output current and voltage were increased by approximately 2.01 and 1.51 times, respectively, in comparison with a conventional Au top electrode without silica spheres. Also, the effect of Au-coated silica spheres on the bending radius of ZnO nanorods was theoretically investigated.

## Background

Energy harvesting technology, capturing ambient waste energy from human movements or machinery vibrations, offers a promising solution for self-powered, wireless, and sustainable operation on various applications such as portable electronic devices, touch sensors, and implanted biosensors [1-3]. Since piezoelectric zinc oxide (ZnO) nanogenerators (NGs) were demonstrated for electric power conversion from mechanical energy in 2006 [4], they have been considered as a key technique for realizing the environment-friendly energy harvesting technology. As an external mechanical force is applied to vertically aligned ZnO nanowires or nanorods using an atomic force microscope (AFM) tip, the positive/negative potential is induced at the stretched/compressed side of ZnO, thus leading to a piezoelectric charge generation [5]. By utilizing this principle of piezoelectric ZnO NGs, over the last decades, there have been considerable efforts to improve the performance and efficiency of piezoelectric ZnO NGs in regard to various types/properties of ZnO nanostructures and surface contact of electrodes. The first one has been achieved by growing ZnO nanowires, nanorods, and nanobelts on the flexible polyethersulfone or polyethylene terephthalate (PET) substrate via a chemical solution method [6,7]. The other one was an alternative way in which zig-zag-shaped or network electrodes (consisting of patterned noble metals, carbon nanotubes, or graphene) were employed as a top electrode to efficiently bend the ZnO nanostructures for transmitting the external mechanical energy as well as possible [8,9]. However, these kinds of top electrodes needed a somewhat sophisticated fabrication process for the preparation of patterned electrodes or synthesis of carbon-based nanomaterials.

On the other hand, one-dimensional (1D) ZnO nanostructures including nanowires or nanorods provide an effective deformation (i.e., stretch and compression) under external mechanical energy due to their high aspect ratio which generates the piezoelectric charges [10]. Additionally, they have been reliably synthesized and vertically integrated on various flexible substrates with ZnO seed coating by hydrothermal or electrochemical deposition (ED) method [11-14]. Particularly, the ED method has many advantages for growing 1D ZnO nanostructures because the electric energy enables a short time process at low temperature [15]. In this work, we prepared ZnO nanorod arrays (NRAs) on an indium tin oxide (ITO)-coated PET substrate (i.e., ITO/PET) using the ED method and fabricated ZnO NRA-based NGs with an efficient top electrode which was obtained by evaporating gold (Au) onto the surface of silica spheres. Herein, the multilayer of silica spheres was facilely deposited on the PET substrate by rolling the colloidal solution of silica spheres.

## Methods

**Figure 1:**
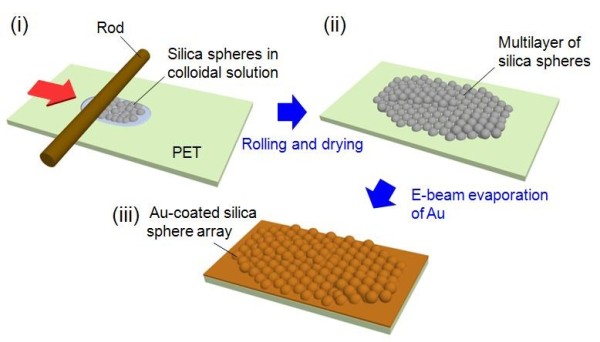
**Schematic diagram for fabrication procedure.** Schematic diagram for the fabrication of the Au-coated silica sphere array as a top electrode of ZnO NRA-based NGs: **(i)** preparation of colloidal solution (i.e., dispersed by silica spheres) on the PET substrate, **(ii)** rolling and drying the colloidal solution, and **(iii)** e-beam evaporation of Au onto the silica sphere array.

Figure 1 shows the schematic diagram for the fabrication of the Au-coated silica sphere array as a top electrode of ZnO NRA-based NGs: (i) preparation of colloidal solution (i.e., dispersed by silica spheres) on the PET substrate, (ii) rolling and drying the colloidal solution, and (iii) e-beam evaporation of Au onto the silica sphere array. Silica spheres were synthesized using a modified Stober process [16]. After the mixture solution with 200 ml of ethanol, 40 ml of ammonia, and 40 ml of de-ionized (DI) water was kept at 60°C, 20 ml of tetraethyl orthosilicate (TEOS) was slowly dropped for 2 h using a burette. Here, all the chemicals were of analytical grade (Sigma-Aldrich, St. Louis, MO, USA). Then, the silica sphere powder was obtained by centrifugation and drying at 70°C. After that, the powder was mixed with ethanol at a concentration of 50 g/l. To increase the viscosity of the colloidal solution, 0.2% weight of poly-4-vinylphenol was added [17]. As shown in Figure 1a, 1 ml of the colloidal solution was dropped on the PET substrate with a size of 3 × 5 cm^2^, and it was spread by slowly pulling the rod with a speed of approximately 1 cm/s. After drying at 60°C for 30 min, Au was coated onto the silica sphere array by e-beam evaporation. In order to ensure adhesion, 20 nm of Cr as an insertion layer was also deposited on the surface of the silica sphere array before deposition of the Au layer.

## Results and discussion

**Figure 2:**
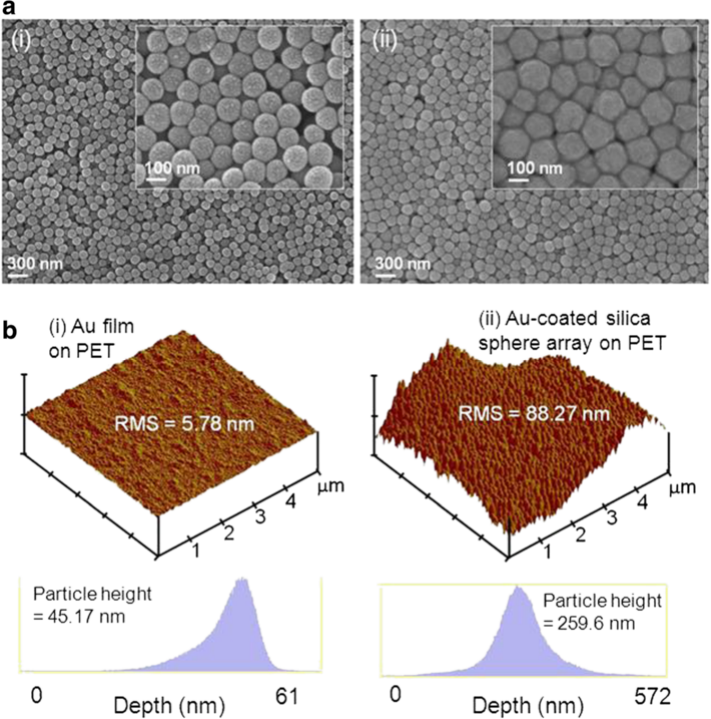
**FE-SEM and AFM images. (a)** FE-SEM images of **(i)** the deposited silica sphere array on the PET substrate and **(ii)** the Au-coated silica sphere array on PET. **(b)** 5 μm × 5 μm scan AFM images and histograms of **(i)** the Au film on the PET substrate and **(ii)** the Au-coated silica sphere on the PET substrate.

Figure 2a shows the field-emission scanning electron microscope (FE-SEM) images of (i) the deposited silica sphere on the PET substrate and (ii) the Au-coated silica sphere array on the PET substrate by e-beam evaporation with a deposition rate of 5 Å/s for 400 s. As shown in the FE-SEM image of Figure 2a (i), the multilayer of silica spheres of approximately 75- to 100-nm diameters was coated on the PET substrate, which could provide a rough surface of the template for Au coating as a top electrode. When Au was deposited on the silica sphere array in Figure 2a (ii), it covered well the whole surface of the silica sphere array with a somewhat thick and angulate morphology. For comparison of the surface roughness in topography, 5 μm × 5 μm scan AFM images and histograms of (i) the Au film on the PET substrate and (ii) the Au-coated silica sphere array on the PET substrate are shown in Figure 2b. As can be seen in the AFM topographic images for each sample, it is clearly observed that the Au-coated silica sphere array had such a rough surface as compared to the surface of the Au film on the PET substrate. From the roughness analysis, the root mean square (RMS) surface roughness of (i) and (ii) were 5.78 and 88.27 nm, respectively. Also, the Au-coated silica sphere array exhibited a high average particle height of 259.6 nm, while the Au film on the PET substrate exhibited a low average particle height of 5.78 nm. This highly rough surface of the Au-coated silica sphere array could lead to a good electrode for efficient bending of ZnO nanorods on NG devices.

**Figure 3:**
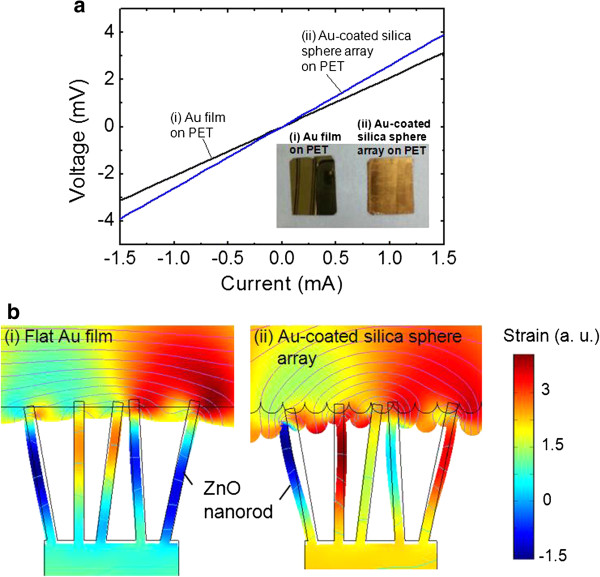
**Electrical characteristics and simulation results. (a)** Measured *I*-*V* curves and **(b)** simulation results of the strain distributions of **(i)** the flat Au film on PET and **(ii)** the Au-coated silica sphere array on PET.

Figure 3 shows (a) the measured *I*-*V* curves and (b) simulation results for the strain distributions of (i) the flat Au film on PET and (ii) the Au-coated silica sphere array on PET. To obtain the sheet resistivity (*R*_s_), the *I*-*V* curves were characterized by a line four-point probe measurement setup with a fixed distance between the probes (1 mm). When the two *I*-*V* curves were compared, the Au-coated silica sphere array exhibited a relatively high slope because the discrete surface of Au somewhat degraded the electric conductivity. Considering the slope and distance, the *R*_s_ values of (i) and (ii) were calculated to be 263.07 × 10^−3^ and 327.54 × 10^−3^ Ω/sq, respectively. Meanwhile, the Au-coated silica sphere array could be expected to yield the efficient bending of ZnO nanorods in ZnO NRA-based NGs as shown in Figure 3b. The strain effects of different surfaces of (i) flat Au and (ii) rough Au on ZnO nanorods were analyzed by the numerical calculation with a commercial software (COMSOL 3.2, stress–strain application mode). Herein, it was assumed that the ZnO nanorods with a size/height of 60 nm/1 μm were bent under an external pushing force of 0.3 kgf/cm [2], and the rough Au has grating structures with a radius of 120 nm, as estimated from the FE-SEM image (in Figure 2a (ii)), for the diameter of Au-coated silica spheres. From the strain distributions of (i) and (ii), it is clear that the bending radius of ZnO nanorods increased more when a pushing force to the NG with roughened Au top electrode was applied. This can be explained by the fact that the curvature of the surface further transmitted the external force to the side of ZnO nanorods. On the contrary, the flat Au transmitted the pushing force to the even surface of ZnO nanorods. For the strain effect of rough Au on ZnO nanorods, it would enhance the performance of ZnO NRA-based NGs with compensation of the slightly increased *R*_s_ of the Au-coated silica sphere array.

**Figure 4:**
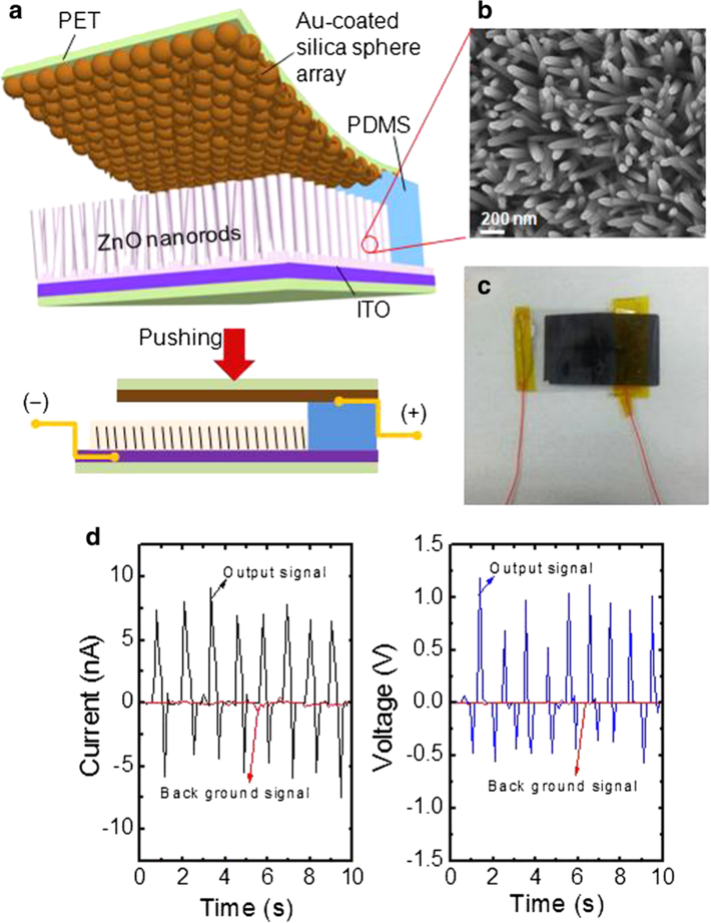
**Schematic diagram and photograph of ZnO NRA-based NG. (a)** Schematic diagram of ZnO NRA-based NG with the Au-coated silica sphere array as a top electrode, **(b)** FE-SEM image of the grown ZnO NRAs on ITO/PET via the ED method, **(c)** photographic image of the fabricated sample, and **(d)** measured output signals in terms of current and voltage for the corresponding sample, in comparison with a background signal.

Figure 4 shows (a) the schematic diagram of the ZnO NRA-based NG with the Au-coated silica sphere array as a top electrode, (b) FE-SEM image of the grown ZnO NRAs on ITO/PET using the ED method, and (c) photographic image of the fabricated sample. In order to fabricate the flexible ZnO NRA-based NG, ITO and Au were used as cathode and anode with PET substrates. The polydimethylsiloxane (PDMS), an elastic soft material, acts as the spacer between the ZnO NRAs and top electrode. This maintained the separation under a leasing pushing force. For the preparation of PDMS, the mixture with base resin and curing agent (weight ratio = 10:1) was poured in a flat petri dish until the thickness reached approximately 8 mm, and cured at 75°C for 2 h. After that, PDMS with a size of 3 × 0.8 cm^2^ was cut and laminated on the exposed surface of ITO/PET (bottom part) as can be seen in Figure 4a. To fix definitely the PDMS between the top electrode and bottom part, a Kapton tape was used for the attachment. After pushing the ZnO NRA-based NG, the bent top electrode is recovered by separating it from ZnO NRAs for the next pushing. Thus, this repeated process enables the rough surface of the Au-coated silica sphere array to compress continuously the ZnO NRAs. During the repeated process, the external pushing force was monitored by using an indicator with load cell (BONGSHIN, Inc. Seoul, South Korea). As shown in Figure 4b, the ZnO NRAs were randomly aligned with an average size/height of about 60 nm/about 1 μm. In the ED process, 20 nm of ZnO seed layer-coated ITO/PET was immersed into the aqueous solution mixture with 20 mM of zinc nitrate hexahydrate and 20 mM of hexamethylenetetramine at approximately 76°C to 78°C. Then, the sample was applied with an external cathodic voltage of −2 V for 1 h by using a simple two-electrode system [7]. The ZnO seed layer was deposited by performing RF magnetron sputtering. As can be seen in Figure 4c, the electric wires were connected to each ITO (cathode) and Au-coated silica sphere array (anode) with the silver paste. Figure 4d shows the measured output signals in terms of current and voltage for the corresponding sample, in comparison with a background signal. Herein, the background signal was obtained by measuring the bare ITO/PET with Au-coated silica sphere array under the same external pushing. It can be clearly observed that the mechanical energy was converted into electrical energy by the induced piezoelectric potential and charge flow between the deformed ZnO NRAs and Au-coated silica sphere array.

**Figure 5:**
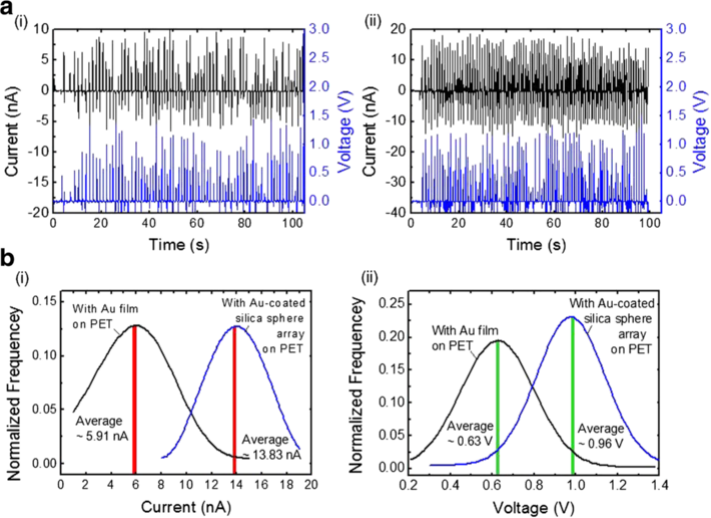
**Measured results of ZnO NRA-based NG. (a)** Measured output current and voltage of the ZnO NRA-based NG with the top electrodes of **(i)** Au film on PET and **(ii)** Au-coated silica sphere array on PET under 0.3 kgf of external pushing force. **(b)** Statistical distributions of the generated output **(i)** current and **(ii)** voltage by Gaussian fits.

Figure 5a shows the measured output current and voltage for the ZnO NRA-based NGs with the top electrodes of (i) Au film on PET and (ii) Au-coated silica sphere array on PET under 0.3 kgf of external pushing force. As a result of measurements, for both ZnO NRA-based NGs, the output currents were induced in positive/negative ways in an AC-type behavior. This might be caused by the fact that the morphology and density of the ZnO nanostructure depend on the induced mode of piezoelectric charge generation [18]. As compared with the (i) and (ii) of Figure 5a, it is clearly observed that the Au-coated silica sphere array yields more increased and regular output current and voltage under 0.3 kgf of external pushing force. When the external pushing force was applied on the top electrode, the highly rough and angulated surface of the Au-coated silica sphere array better transmitted the mechanical force to the ZnO NRAs as expected from the simulation result of Figure 3b. In order to estimate the performance enhancement of samples, the statistical distributions were figured out by Gaussian fits from the measured values of the generated output (i) current and (ii) voltage in Figure 5b. Considering the averaged values, the output current and voltage were increased by about 2.01 and 1.51 times. This confirms that the Au-coated silica sphere array played the role of an efficient top electrode on the ZnO NRA-based NGs.

## Conclusion

We successfully fabricated the efficient top electrode for ZnO NRA-based NGs by incorporating the Au-coated silica sphere array on the PET substrate. When Au was deposited onto the multilayer of silica spheres, it formed as a highly rough surface with angulated morphology. By computational simulations for the strain distribution when bending ZnO nanorods, the rough surface of Au-coated silica sphere array could be expected to further increase the bending radius under an external pushing force. For an experimental analysis, the NGs were fabricated with ZnO NRAs on ITO/PET via the ED method and different top electrodes (i.e., Au film on PET and Au-coated silica sphere array on PET). Under an external pushing force of 0.3 kgf, the Au-coated silica sphere array contributed to the improvement in output current and voltage by about 2.01 and 1.51 times with regular curves. From these results, the Au-coated silica sphere array could be useful for an efficient top electrode in various ZnO nanostructure-based piezoelectric NG applications.

## Competing interests

The authors declare that they have no competing interests.

## Authors’ contributions

YHK designed and analyzed the NRA-based NGs with the Au-coated silica sphere array as an efficient top electrode. GN assisted in the chemical synthesis and measurements (FE-SEM and AFM). JSY supervised the conceptual framework and drafted the manuscript. All authors read and approved the final manuscript.
